# PanicRoom: a virtual reality-based Pavlovian fear conditioning paradigm

**DOI:** 10.3389/fpsyg.2024.1432141

**Published:** 2024-10-04

**Authors:** Chiara Lucifora, Aldo Gangemi, Giovanni D’Italia, Laura Culicetto, Francesca Ferraioli, Giorgio Mario Grasso, Carmelo Mario Vicario

**Affiliations:** ^1^Department of Philosophy, Alma Mater Studiorum University of Bologna, Bologna, Italy; ^2^Institute of Cognitive Sciences and Technologies, National Research Council, Rome, Italy; ^3^Department of Cognitive Science, University of Messina, Messina, Italy

**Keywords:** virtual reality, fear conditioning, experimental paradigm, Pavlovian fear conditioning task in virtual reality, Pavlovian (classical) conditioning

## Abstract

**Introduction:**

Pavlovian fear conditioning is an experimental paradigm used to study the acquisition and extinction of fear responses and the various aspects of fear and anxiety. We developed a virtual reality (VR) version of this paradigm to leverage the benefits of virtual reality, such as ecological validity, standardization, safety, and therapeutic applications. Our objective was to create an open-source and immersive environment for studying fear-related responses using Unity Engine 3D and the Oculus Rift device.

**Methods:**

In this virtual environment, the participants encountered a monster screaming at 100 dB approaching them as the fear-inducing stimulus (unconditioned stimulus or US). Our protocol included three sessions: habituation, acquisition, and extinction, with two stimuli associated with different doors (blue vs. red). The blue door (CS+) was linked to the US, while the red door (CS−) was the control. We tested this VR paradigm on 84 young participants, recording their skin conductance response (SCRs) and fear stimulus ratings (FSRs) on a 10-point Likert scale.

**Results:**

The findings showed significantly higher SCRs and FSRs for CS+ as compared to CS− during the acquisition phase and higher SCRs and FSRs for CS+ during the acquisition phase as compared to the habituation and extinction sessions.

**Discussion and conclusions:**

These results supported the reliability of the protocol for studying fear and anxiety-related conditions.

## 1 Introduction

Based on Ekman’s model of emotions (Ekman, 1999), fear is one of the six fundamental and universally experienced human emotions, alongside happiness, sadness, anger, surprise, and disgust. Fear can be defined as a central state of an organism that depends on context (external stimuli) and can lead to specific behaviors, such as fight or flight (Adolphs, 2013).

Fear conditioning is a classic psychological paradigm that allows for the study of fear acquisition and extinction using neutral and fear-inducing stimuli (Lonsdorf and Merz, 2017). This protocol is widely used in research on anxiety and related disorders, such as post-traumatic stress disorder (Marković et al., 2021; Fullana et al., 2016; McDannald, 2023), and facilitates the examination of how environmental and intrinsic factors influence affective learning processes (Lucifora et al., 2022). During the acquisition phase, a neutral stimulus (e.g., a tone or geometric figure) is repeatedly paired with an aversive stimulus (e.g., a shock). Over time, the neutral stimulus alone begins to elicit a fear response (e.g., increased electrodermal activity), demonstrating the learned association between the neutral stimulus and the aversive event. During extinction, the previously conditioned stimulus is presented without the aversive stimulus. Over repeated trials, the conditioned fear response gradually decreases and eventually disappears. This indicates that the association between the conditioned stimulus and the aversive event is weakened or eliminated.

To date, this type of paradigm is the most widely used in the study of fear and anxiety (Fullana et al., 2016). However, some important limitations characterize current protocols:

1. As explained by Löw et al. (2015), in the classical fear conditioning paradigm, the fearful stimulus typically prompts a defensive freeze rather than active avoidance of the threat. Commonly, fear stimuli involve delivering an electrical shock to the participant’s skin (Wiech and Tracey, 2013). The intensity of the stimulus varies based on individual perception, making it challenging to create a universal fearful stimulus. This results in significant individual variability, known as a “weak situation” (Lonsdorf et al., 2017). To address these limitations, it is crucial to design a more effective experimental protocol that elicits consistent responses across all participants (Lissek et al., 2006).
2. There is no consensus on the calibration of a fear stimulus (Duncan et al., 1989). Typically, it relies on participants’ self-evaluations. For example, in fear conditioning studies, the intensity is often set to a level perceived as “dislike,” whereas in pain-related fear conditioning, it may be calibrated to a level perceived as “pain not easily tolerated” (Lonsdorf et al., 2017). The absence of a standardized calibration method poses a significant problem, affecting the salience and acquisition of fear. As stated by Lissek et al. (2008), if a fear stimulus is incorrectly perceived as harmful, generalization may not occur as intended. This means its function of enabling participants to appropriately respond to new stimuli based on past experiences does not manifest (Lissek et al., 2008).
3. Another common issue in fear conditioning studies is the exclusion of participants based on their performance. There are no specific guidelines or criteria defining who has acquired or extinguished fear, such as a general cut-off in explicit and/or implicit measures. This lack of standardization raises concerns about the replicability and comparability of the studies (Lonsdorf et al., 2017). This issue is part of a broader problem related to the heterogeneity in the operationalization of fear conditioning studies, as highlighted by Lonsdorf et al. (2019).
4. Finally, there is an issue related to context as the environment in which the experiment occurs can influence both the acquisition and extinction phases. Research has shown that various contextual details, such as room lighting, scents, and computer backgrounds, may inadvertently influence the results (Maren et al., 2013; Urcelay and Miller, 2014).

Virtual reality (VR) can help address these issues by standardizing contextual elements. Generally, VR allows for the creation of safe and ecological environments, within which behaviors that are difficult to assess in real life can be studied (Vicario and Martino, 2022; Ferraioli et al., 2024). This capability is particularly relevant for fear conditioning paradigms.

The study conducted by Gromer et al. (2019) demonstrated a bidirectional relationship between presence and fear, showing that higher fear ratings correlate with higher presence ratings and vice versa. This relationship was also confirmed by studies on social phobia, such as those by Bouchard et al., 2008 and Peperkorn et al., 2015. In this context, the strong relationship between presence and fear in VR can help researchers avoid issue 1 (the universality of fearful stimuli) by ensuring that fear responses manifest across a wide range of participants. In addition, it can improve the management of the exclusion criteria (issue 3). Furthermore, the ability of VR to present vivid and realistic threats, creating an illusion of plausibility, addresses issue 2 (i.e., calibration of fear stimuli). Unlike physical stimuli such as electrical shocks, VR stimuli do not require specific calibration to the user’s body, which enables researchers to study fear without causing pain. For example, Lin (2017) demonstrated that a horror VR game featuring zombies is an excellent method for studying fear response and coping strategies. Finally, the study by Kroes et al. (2017) demonstrated that virtual contexts allow researchers to easily record participants’ subjective perception of threat, their threat-conditioned defensive response, and their explicit memory of the threat. This is supported by the review work of Andreatta and Pauli (2021), which showed that virtual contexts can effectively induce fear and anxiety in participants, thereby addressing issue 4 mentioned above.

Overall, VR provides a valid tool for studying fear conditioning due to its ability to create a sense of presence, induce realistic responses, and simulate environments in a more ecological manner. However, currently available protocols for fear conditioning do not include and/or provide evidence of psychophysiological modulation in response to exposure to conditioned stimuli (Quezada-Scholz et al., 2022). This represents a major limitation as the modulation of psychophysiological measures such as skin conductance response (SCR) is crucial for verifying effective fear conditioning through a Pavlovian protocol (e.g., Miller et al., 2023; Bach et al., 2018).

In this article, we present the results of a new, open-source, VR-based protocol called “*The PanicRoom.”* Its effectiveness for fear conditioning is demonstrated by examining both behavioral (i.e., by collecting the fear stimulus rating—FSR) and psychophysiological (by collecting the skin conductance response—SCR) measures.

## 2 Virtual reality paradigm: “the PanicRoom”

We created a 3D environment in virtual reality using Unity Engine 3D and Oculus Rift as the VR Headset. Our experimental setup included the Oculus Rift device for virtual reality presentation, featuring two Pentile OLED displays with a resolution of 1,080 × 1,200 per eye, a 90 Hz refresh rate, and a 110 ° field of view. The device also includes features for rotation and position tracking, as well as integrated headphones that provide a 3D sound effect (Grasso et al., 2019). A graphics workstation equipped with an NVIDIA Titan X graphics card was used to run the simulation, ensuring a uniform high-resolution rendering of the virtual environment projected to the VR headset (Lucifora et al., 2022).

For the virtual environment, we acquired the “*Door Free Pack Aferrar”* for the general setting and the “*True Horror – Crawler”* package for the monster from the Unity Asset Store. Our environment consisted of a simple room with two doors and a floor. The participant was positioned in front of the doors (CS+ and CS−), and the virtual camera was set at a height that gave the user the perception of being seated. Our unconditioned stimulus (US) involved a virtual monster that emitted a scream, produced by a mono mp3 audio file, with the volume set to 100db. Based on previous studies (Lau et al., 2011; Glenn et al., 2012; Shechner et al., 2015), we chose a screaming lady sound for this purpose. The monster was programmed to jump outside the door, getting very close to where the participant was standing. For all the Unity 3D scripts, we used C# as the programming language.

Following the Pavlovian model, our VR paradigm comprised three sessions: habituation, acquisition, and extinction. During these sessions, the two doors were presented randomly.

**Figure 1:**
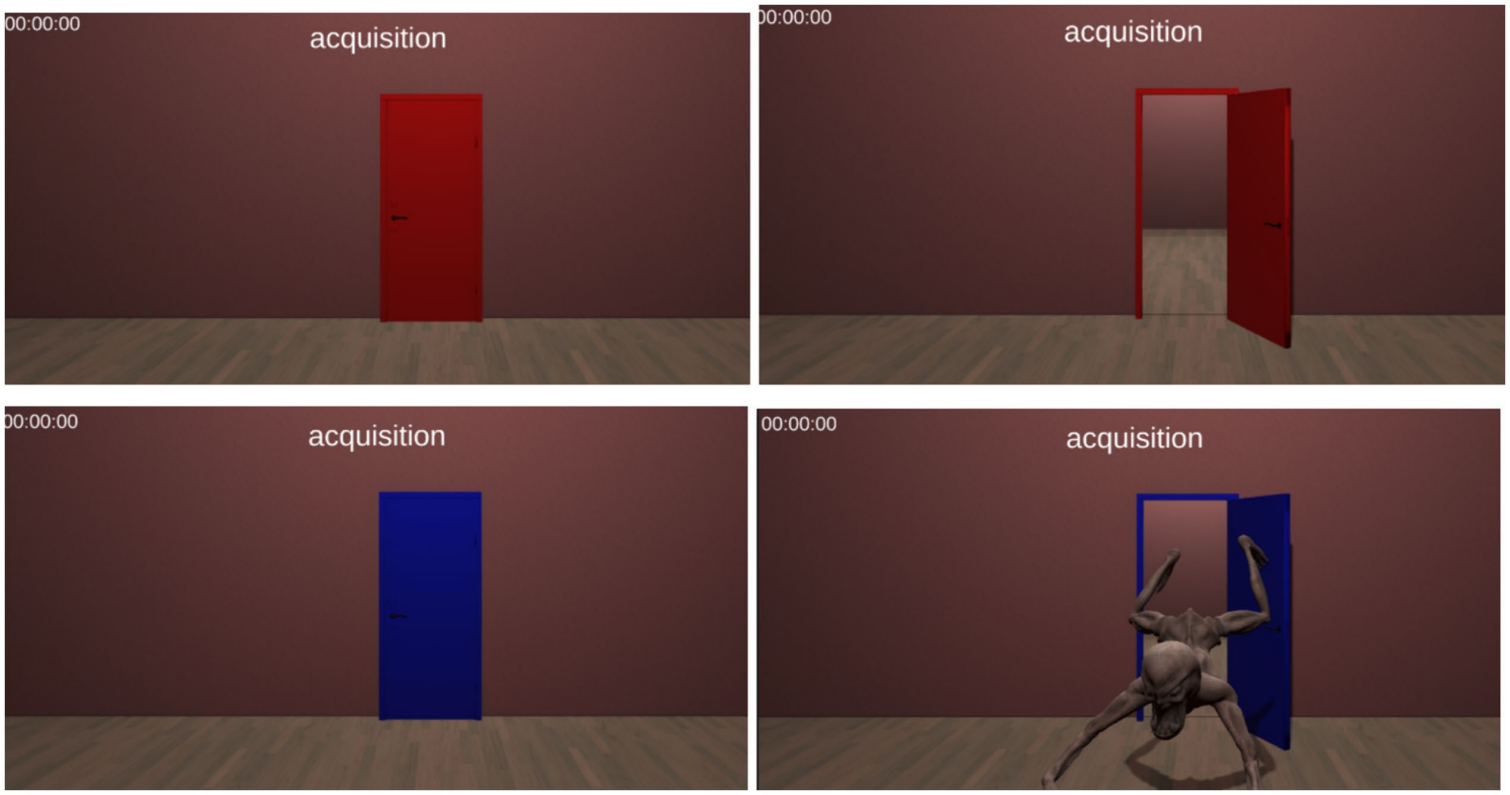
A screenshot of the acquisition phase in our PanicRoom paradigm. The upper panels depict the CS− (red door), while the lower panels depict the CS+ with the US (blue door with the fearful stimulus).

1. The habituation phase lasted 4 min and involved presenting two differently colored doors (eight trials in total: four CS+ blue doors and four CS− red doors). Each door was displayed for 12 s in a random order. There was a 3-s interval between each trial, with the first block lasting approximately 2 min. During the habituation phase, each door was presented individually; it opened 9 s after its appearance and remained open for an additional 3 s. No stimuli were presented while the door was open in this phase.
2. The fear acquisition phase started after a short pause of 60 s. This phase included 10 CS+ trials, in which eight blue doors (80%) were immediately followed by the leaping monster as part of the partial reinforcement program. In addition, there were 10 CS− trials, during which no stimulus was present once the red door was opened. Each trial lasted for 12 s, which included 9 s with the door closed and 3 s with the door open (Figure 1).
3. The extinction phase started after a 5-min break, during which the user was instructed to exit the virtual environment. Extinction training involved 10 CS+ trials and 10 CS− trials, similar to the habituation phase, without any stimulus presented when the doors were open. This phase can be repeated 24 h later, referred to as the “recall phase.” Although many studies opt for immediate extinction training, the recall phase holds clinical relevance as it allows for memory consolidation before fear extinction (Lonsdorf et al., 2017). The number of trials in our paradigm is standard, matching those used in other studies with different paradigms for fear conditioning (e.g., Zuj et al., 2016; Zuj et al., 2017a,b).

**Figure 2:**
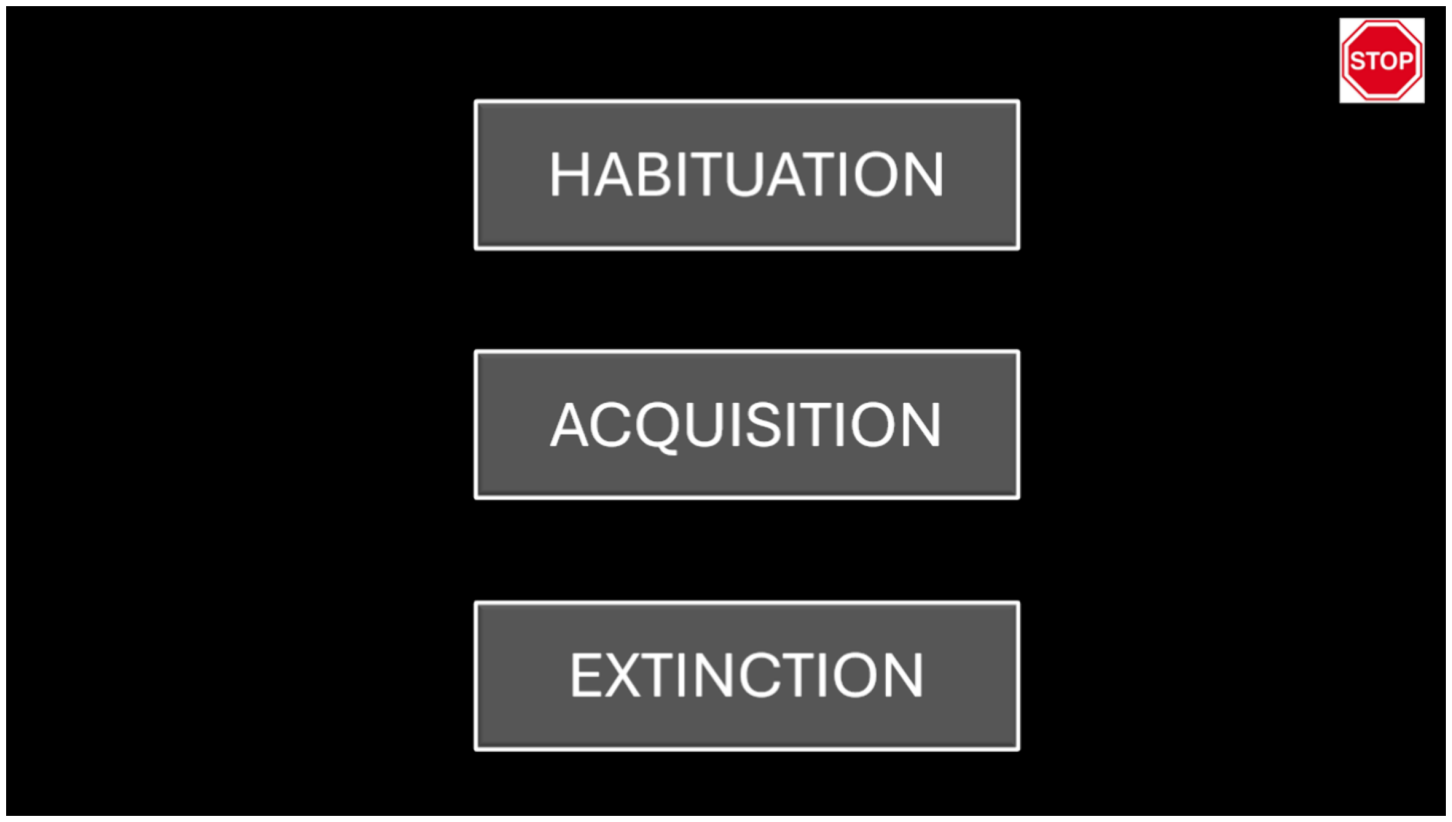
This image displays our graphical user interface, which allowed the researcher to easily select among the three phases (habituation, acquisition, and extinction). An emergency button, located in the top-right corner, enabled the researcher to stop the simulation immediately.

Our software interface allowed the researcher to easily select among the three phases by clicking a button on the graphical user interface. Different buttons corresponded to the different phases of the protocol. In addition, we implemented an emergency button that could stop the simulation immediately (see Figure 2).

**Figure 3:**
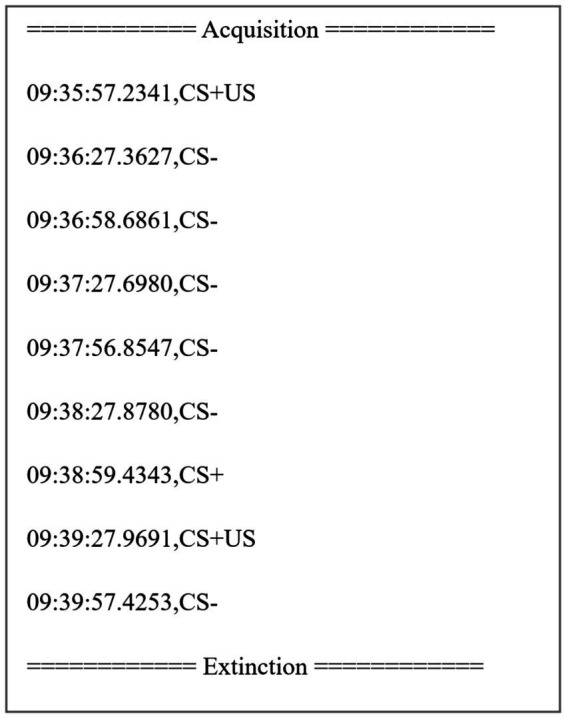
This image displays an example of the stimuli timing information saved by the system in a txt file. Our system was programmed to record the time in the format hh:mm:ss.ms, followed by the conditioned stimulus (CS−, CS+, or CS + US).

Furthermore, our application included a timekeeper for the experiment, which was visible only to the researcher on the desktop screen. It saved timing information in an external text file, allowing the researcher to track the occurrences of CS+, CS−, and CS + US for all phases. Figure 3 shows an excerpt from a typical text file.

## 3 Model representation

**Figure 4:**
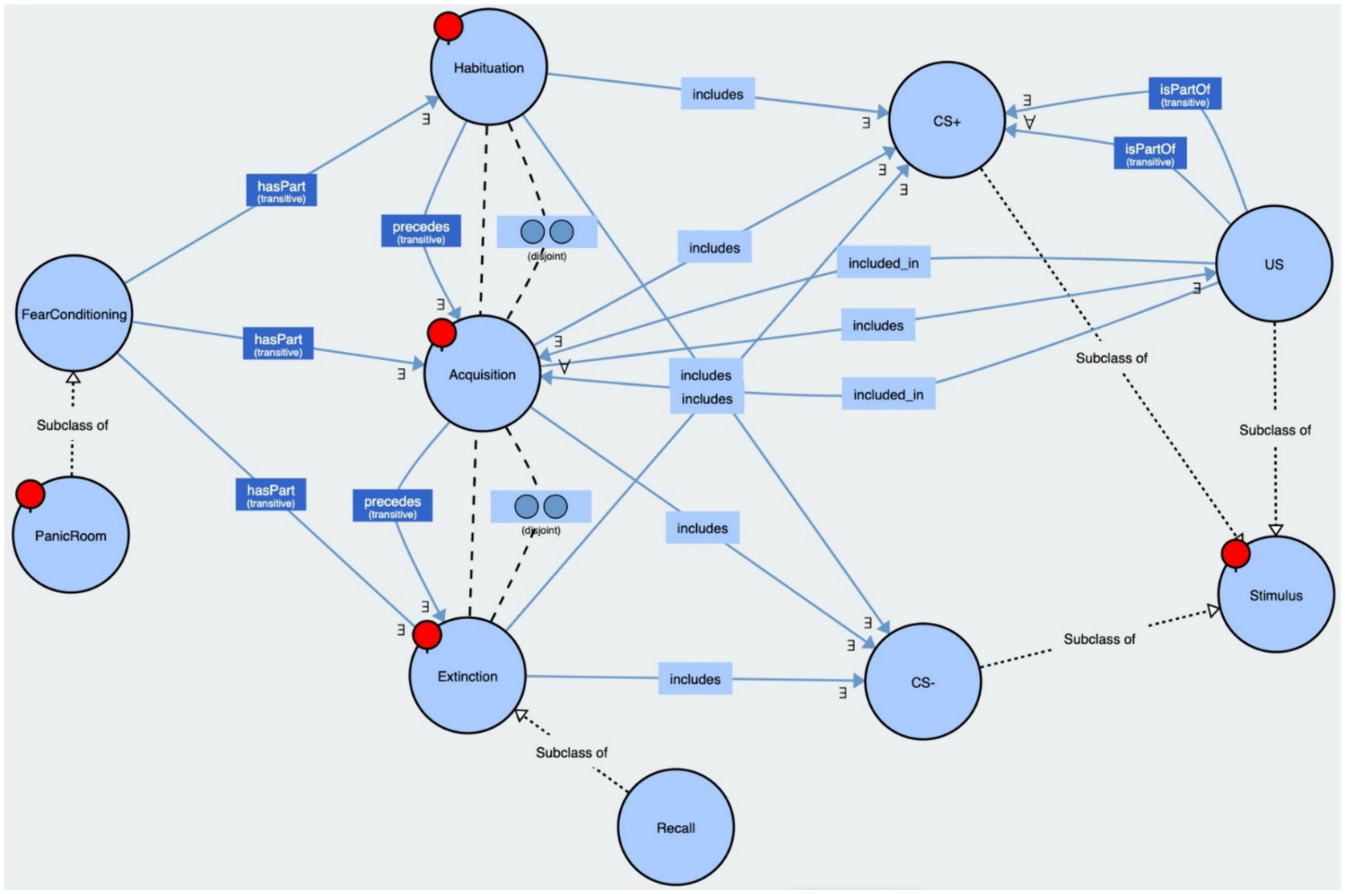
This figure displays our PanicRoom ontology. The bubbles represent the classes, the solid arrows indicate the relations between the classes along with their associated cardinalities, and the dashed arrows denote the subclasses or disjoint classes. Both the ontology and the executable are available on GitHub (https://github.com/aldogangemi/panicroom).

We created an ontology of our paradigm, implemented in OWL 2, to explain the relations among the classes of experimental phases and stimuli based on the classical Pavlovian Fear Conditioning Paradigm. Our PanicRoom VR paradigm encompassed the three fear conditioning phases and the generic structure of the stimuli (CS+, CS−, US). These phases were modeled as disjoint classes within the paradigm, including the associated stimuli. Habituation preceded acquisition, which was followed by extinction and potentially a recall phase. The US stimulus was included only in the acquisition phase and associated with some randomly selected CS+. See Figure 4 for reference.

## 4 Validation test

### 4.1 Participants

A total of 85 participants were initially recruited through online advertisements. One participant was excluded due to technical problems during the recording of the SCR measures, resulting in a total sample of 84 participants (30 male participants), with a mean age of 22 ± 2.95 years. Informed consent was obtained from all participants before inclusion, and the protocol was approved by the local ethics committee of the Department of Cognitive, Psychological, Pedagogical, and Cultural Studies (Approval n. COSPECS_4_2021; COSPECS_07_2022), University of Messina, Italy. The experimental procedures were conducted in accordance with the principles of the Declaration of Helsinki (1964) and its subsequent updates (World Medical Association, 2013).

#### 4.1.1 Skin conductance response

The skin conductance response (SCR) was measured using eSense (Mindfield Biosystems Inc., Berlin, Germany) with a MEIZU M5C M710H device, featuring electrodes that were attached to the middle and index fingers of the participants with Velcro straps. The electrodes were connected to the eSense device with an audio-type connection input. The eSense device acquired data at a sampling rate of 5 Hz, which were exported from the eSense-connected PC through email in a csv format. In accordance with previous studies (Dawson et al., 2007; Zuj et al., 2017a,b), the SCR for CS+ and CS− was calculated in microSiemens (μS) by subtracting the mean conductance over the 2 s before stimulus onset (baseline) from the peak conductance level acquired during the 9 s stimulus presentation (closed door).

#### 4.1.2 Fear stimulus rating

At the end of each session, the participants were asked to rate “*how scary the presented stimuli were* (i.e., red and blue doors).” This approach helped avoid distracting the participants by collecting this information after each trial. Fear stimulus ratings (FSRs) were provided using a 10-point Likert scale, where 1 indicated “not scary at all” and 10 indicated “extremely scary.”

### 4.2 Procedure

After the participants provided informed consent, they were connected to the GSR Amp (eSense) and two ring-shaped skin conductance electrodes were placed over the middle and index fingers of their right hand. The virtual reality helmet (Oculus Rift) was then placed on the head, and then, the fear conditioning/extinction task was conducted as described above. At the end of the experiment, the participants were debriefed. Overall, the procedure took about 30 min to complete.

### 4.3 Data analysis

Statistical analyses were conducted to determine whether the current protocol effectively prompted fear conditioning and fear extinction in our experimental participants. Comparisons were made using the exposure to the fear conditioning/extinction task as the independent variable, while the SCR trial-by-trial sample mean and the FSR overall sample mean scores served as the dependent variables.

The SCR amplitude was determined offline by subtracting the baseline SCR, measured 2 s before the CS presentation, from the highest skin conductance level during each CS presentation. This calculation was performed for the individual acquired data of each participant. For the SCR data analysis, a square root transformation was applied to reduce variability, in accordance with previous studies (e.g., Vicario et al., 2020; Ney et al., 2021; Vicario et al., 2023).

The SCR and FSR data were analyzed separately using 3 (Session: habituation, acquisition, extinction) × 2 (Stimulus: CS+, CS–) repeated measures ANOVAs. Partial-eta squared (*η_p_^2^*) was calculated as an effect size. Post-hoc analyses were conducted using the Scheffé test in case of significant results from the ANOVAs. This test provides a robust method for examining specific group differences while controlling for Type I error (the probability of incorrectly rejecting a true null hypothesis—false positive). In addition, we performed a sensitivity analysis to assess whether the study design was sufficiently powered to detect the expected result. The results suggested that if the true effect size for the variable of interest is 0.309 or larger, then the study design (with 84 participants, a p-level of 0.05, and a power of 80) is adequately powered to detect it. A critical alpha level of 0.05 served as the significant threshold. The statistical analyses were performed using STATISTICA (StatSoft. Inc., Tulsa, OK, United States) version 7.0 and G*Power.

## 5 Results

### 5.1 Skin conductance response

**Figure 5:**
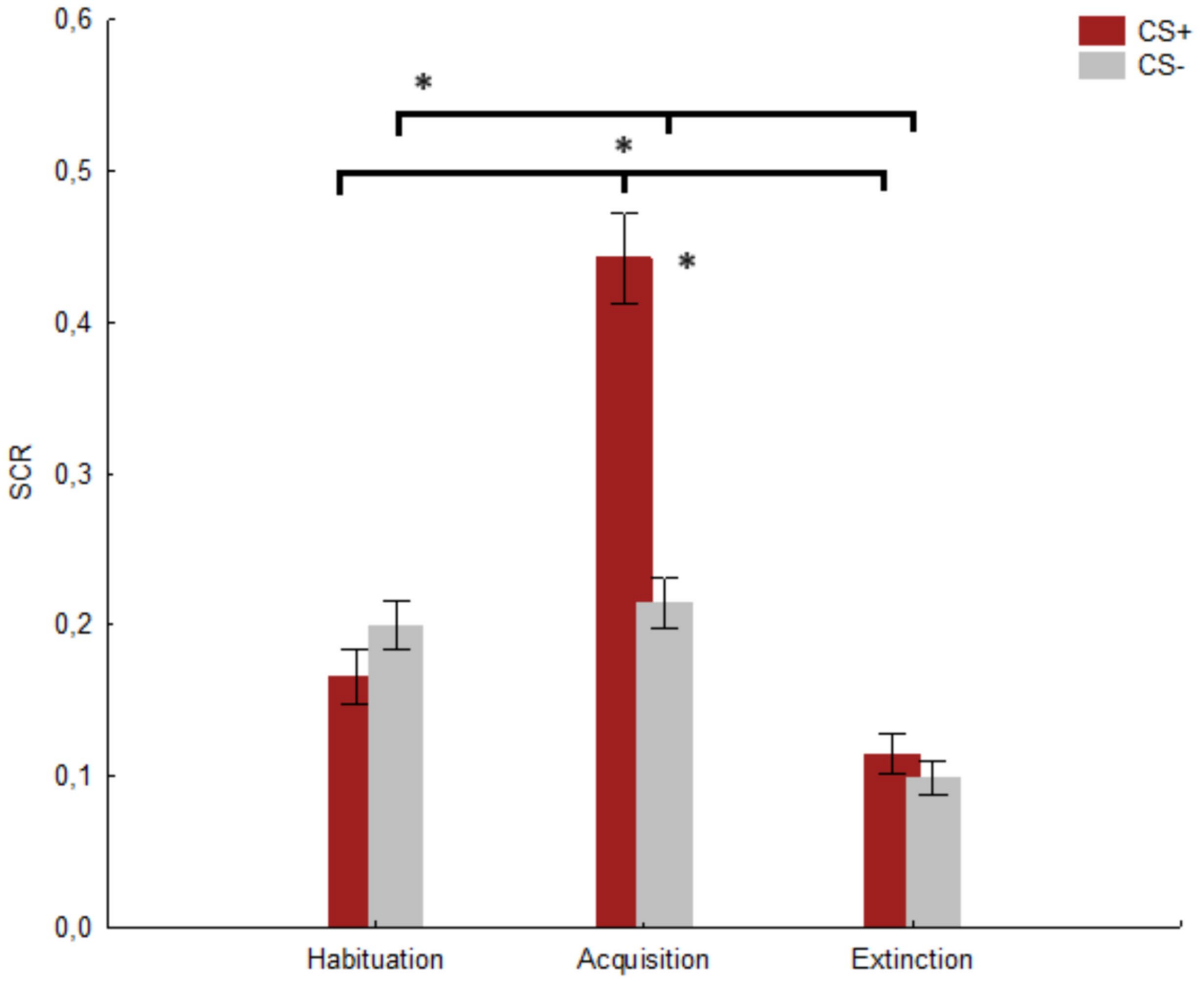
The figure shows the mean SCR for CS+ and CS− in the three sessions. * indicates significant results (*p* ≤ 0.001). Vertical bars denote +/− standard errors of means.

Due to technical problems during the recording of the SCR measures, one participant was removed from the dataset. A significant main effect of the factor Session was found [*F*(2, 164) = 72.23, *p* < 0.001, *h_p_^2^* = 0.468; *Observed power*: 1.000]. The post-hoc comparisons showed a significant difference (*p* < 0.001) between habituation (*M* = 0.182, *SE* = 0.015) and acquisition (*M* = 0.328, *SE* = 0.021), as well as (*p* < 0.001) between acquisition and extinction (*M* = 0.106, *SE* = 0.010) and habituation and extinction (*p* < 0.001). Furthermore, the main effect of the factor Stimulus was significant [*F*(1, 82) = 41.04, *p* < 0.001, *h_p_^2^* = 0.333; *Observed power*: 0.999], with a higher score (*M* = 0.241, *SE* = 0.015) in response to CS+ compared to CS− (*M* = 0.170, *SE* = 0.011). Finally, a significant Stimulus x Session interaction was revealed [*F*(2, 164) = 73.15, *p* < 0.001, *h_p_^2^* = 0.471, *Observed power*: 1.000]. The post-hoc comparisons showed a significant difference (*p* < 0.001) between CS+ (*M* = 0.442, *SE* = 0.029) and CS− (*M* = 0.214, *SE* = 0.016) in the acquisition session. Moreover, a significant difference (*p* < 0.001) for CS+ was found between habituation (*M* = 0.165, *SE* = 0.018) and acquisition (*M* = 0.442, *SE* = 0.029) and (*p* < 0.001) between acquisition and extinction (*M* = 0.114, *SE* = 0.0218). Finally, a significant difference (*p* < 0.001) was found for CS− between habituation (*M* = 0.199, *SE* = 0.016) and extinction (*M* = 0.098, *SE* = 0.010) and (*p* < 0.001) between acquisition (*M* = 0.214, *SE* = 0.016) and extinction. No significant difference was found between CS+ and CS− for habituation (*p* = 0.520) and extinction (*p* = 0.963), and no significant difference was found for CS− between habituation and acquisition (*p* = 0.969) c.f. Figure 5.

### 5.2 Fear stimulus rating

**Figure 6:**
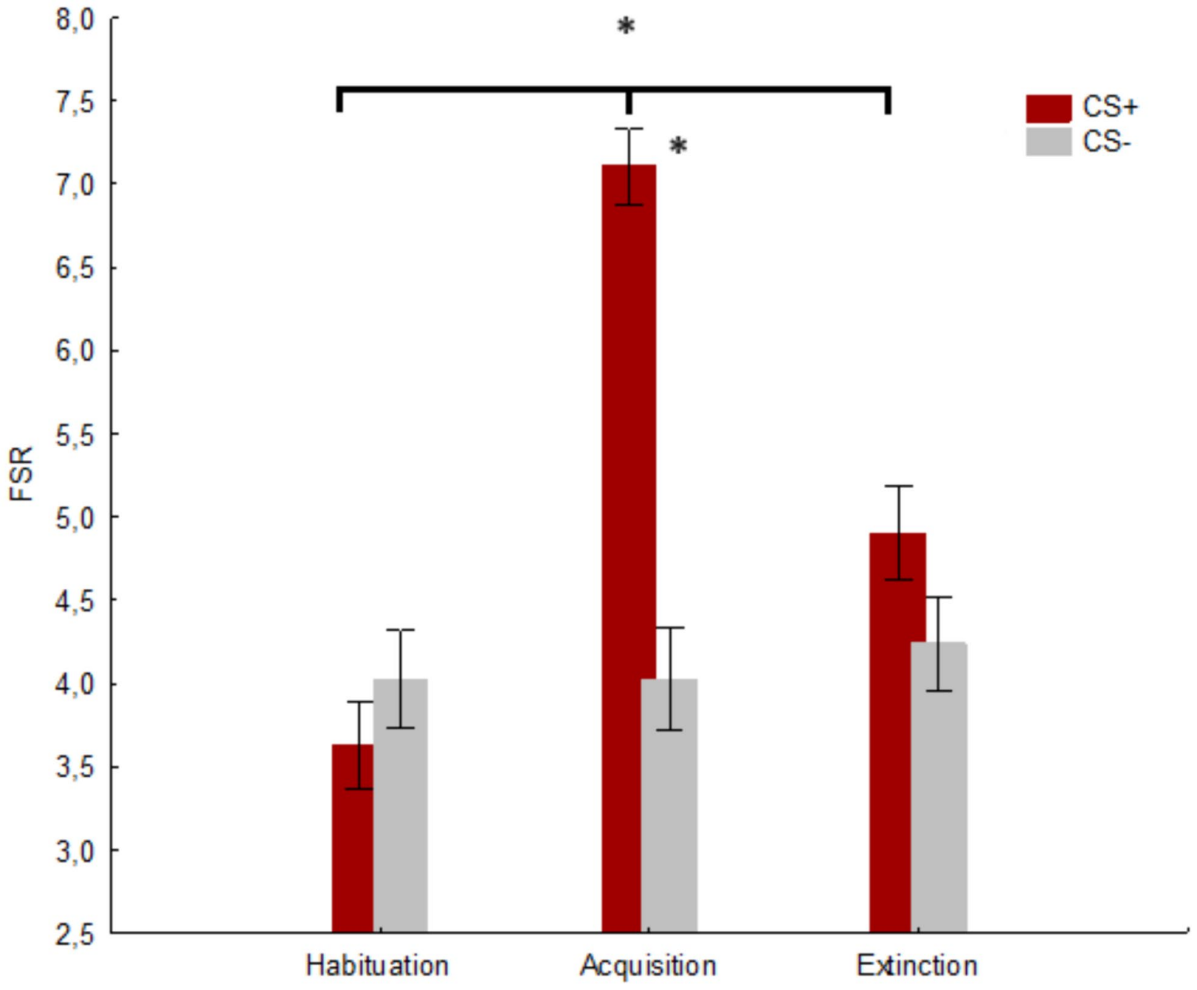
The figure shows the mean fear stimulus ratings (FSRs) for CS+ and CS− in the three sessions. * indicates significant results (*p* ≤ 0.001). Vertical bars denote +/− standard errors of means.

A significant main effect of the factor Session was found [*F*(2, 166) = 32.25, *p* < 0.001, *h_p_^2^* = 0.279; *Observed power*: 1.000]. The post-hoc comparisons showed a significant difference (*p* < 0.001) between habituation (*M* = 3.827, *SE* = 0.257) and acquisition (*M* = 5.655, *SE* = 0.214), as well as (*p* < 0.001) between acquisition and extinction (*M* = 4.571, *SE* = 0.265) and habituation and extinction (*p* = 0.003). Furthermore, the main effect of the factor Stimulus was significant [*F*(1, 83) = 43.81, *p* < 0.001, *h_p_^2^* = 0.345; *Observed power*: 0.999)], with a higher score (*M* = 5.214, *SE* = 0.201) in response to CS+ compared to CS− (*M* = 4.095, *SE* = 0.253). Finally, a significant Stimulus x Session interaction was revealed [*F*(2, 166) = 60.85, *p* < 0.001, *h_p_^2^* = 0.423, *Observed power*: 1.000)]. The post-hoc comparisons showed a significant difference (*p* < 0.001) between CS+ (*M* = 7.107, *SE* = 0.229) and CS− (*M* = 4.023, *SE* = 0.308) in the acquisition session. Moreover, a significant difference for CS+ was found between habituation and acquisition (*p* < 0.001) and extinction (*p* < 0.001), as well as between acquisition and extinction (*p* < 0.001). No significant difference was found between CS+ and CS− in the habituation (*p* = 0.706) and extinction (*p* = 0.136) sessions. No difference was found for CS− between the three sessions (*p* ≥ 0.706) c.f. Figure 6.

## 6 Discussion

Virtual reality is a promising tool for creating a more ecological and experimental paradigm in fear conditioning studies. Recent studies (Baas et al., 2004; Kroes et al., 2017) have highlighted the importance of a virtual context for improving fear and anxiety learning, using both explicit and implicit measures. However, it is important to note that some studies have not provided robust evidence of its effectiveness. For example, in the study by Quezada-Scholz et al. (2022), the conditioned response was confirmed solely through explicit measures, such as subjective anxiety ratings, rather than by physiological measures related to the startle reflex.

In our study, we assessed the effectiveness of an immersive VR-based Pavlovian fear conditioning paradigm by measuring both psychophysiological (SCR) and behavioral (FSR) responses across three prototypical sessions (i.e., habituation, acquisition, and extinction). The results validated the expected fear conditioning–-extinction pattern for both explicit (FSR) and implicit (SCR) measures. As predicted, we found higher SCRs and FSRs for CS+ compared to CS− during the acquisition session, with no differences observed during the habituation and extinction sessions. These findings suggested that the fear conditioning occurred as expected; this was further supported by the higher SCR and FSR for CS+ in the acquisition session compared to the habituation and extinction sessions. However, the absence of any difference in the SCR and FSR between CS+ and CS− during the habituation and extinction sessions suggested that the adopted stimuli (as anticipated) were initially perceived as neutral during the habituation session and were no longer perceived as threatening during the extinction session. Importantly, the evidence that our protocol also modulated the psychophysiological response (SCR), in contrast to previous protocols (Quezada-Scholz et al., 2022), suggested that it was more effective in inducing fear conditioning. The SCR is considered a key measure for verifying effective fear conditioning within a Pavlovian paradigm (e.g., Miller et al., 2023; Bach et al., 2018). Importantly, while we did not conduct an *a priori* analysis for our sample size, the sensitivity analysis indicated that our sample size was sufficient to reliably detect medium to large effects. The effect sizes for the SCR (0.471) and FSR (0.423) both exceeded the threshold of 0.309, confirming that our sample size was adequate. Furthermore, the observed power score associated with the relevant results suggested a very high likelihood of detecting a true effect, should one exist (Cohen, 1992; Button et al., 2013).

In statistical terms, power refers to the probability of rejecting a null hypothesis when it is false. An observed power of 0.99 or higher (as in our case) indicates that a study is very well powered and has a very minimal probability of committing a Type II error (false negative), suggesting that the study is highly sensitive to detecting the effect being investigated (Button et al., 2013).

Our protocol enhances the classical non-VR-based fear conditioning paradigm by addressing several limitations and introducing new advantages. First, it effectively elicits a universal fear response among participants (issue 1), as evidenced by the high FSR score reported by the participants; it reduces issues related to the calibration of fear stimuli (issue 2) and the subsequent exclusion of participants (issue 3). In addition, our paradigm utilizes a simple, standardized context (a room with just two doors), minimizing the potential for undesirable influences from external factors (issue 4). Finally, our protocol ensures precise replication of trials in terms of duration and stimulus presentation, thus addressing concerns associated with the “replication crisis” (Stroebe and Strack, 2014). Furthermore, our protocol offers numerous advantages over classical fear conditioning paradigms, particularly those employing electrical shocks. It enhances the ecological validity of the classical paradigms by allowing the study of fear responses in scenarios that resemble those encountered in everyday life. This improvement leads to enhanced validity and interpretation, which may be limited in the paradigms using electrical shocks due to factors such as individual differences in pain sensitivity. In this sense, our protocol does not involve pain, unlike those using electrical stimulation as the US.

In conclusion, our open-source, VR-based Pavlovian fear conditioning paradigm represents a novel, powerful, and versatile tool for studying fear and anxiety. It offers researchers greater control, increased realism, and enhanced flexibility compared to classical fear conditioning methods.

## Data Availability

Publicly available datasets were analyzed in this study. This data can be found here: https://github.com/aldogangemi/panicroom.
